# Overview of Services for Autism Spectrum Disorders (ASD) in Low- and Middle-Income Countries (LMICs) and among Immigrants and Minority Groups in High-Income Countries (HICs)

**DOI:** 10.3390/brainsci12121682

**Published:** 2022-12-08

**Authors:** Sayyed Ali Samadi

**Affiliations:** Institute of Nursing and Health Research, Ulster University, Belfast BT15 1ED, UK; s.samadi@ulster.ac.uk

Autism spectrum disorder (ASD) prevalence is rising [1] at different paces. The reported prevalence currently lies between 0.9% and 1.5% [2]. In affluent and high-income countries, a higher speed is reported compared to a slower pace in developing countries [3]. A recent worldwide ASD prevalence estimate of 0.6% falls far below estimates for Western developed societies. This reported difference probably reflects an inability to diagnose due to the shortage of available diagnostic services rather than a natural worldwide variation in the incidence of ASD in its different forms [4]. There are various contributing factors to the reported difference. It reflects inequality in broadening the diagnosis services and different levels of awareness among countries and multiple degrees of endeavor for identification among children primarily. Hence, the increasing international wealth of information on ASD is based mainly on numerous studies conducted in developed, affluent countries [5]. As de Leeuw et al. [6] indicated, “Autism research is heavily skewed towards western high-income countries”.

More attention should be considered to the presence of similarities and differences in the representation and the influence of ASD in different world regions and between different cultures and minority groups. Still, the available wealth of data remains limited in low-and-middle-income countries (LMICs) and among immigrants and minority groups in high-income countries (HICs). In many ways, there is considerable overlap between the situation for individuals with ASD in LMICs and HICs [5]. There are similar challenges for this group of individuals and their family members and caregivers in both country groups. Hence, there are additional cultural, political, and economic challenges pertaining to LMICs [4]. There are different aspects of ASD that have been understudied, and investigating their impacts on particular groups of individuals is missed in LMICs and HICs. It seems that healthcare policymakers were not entirely convinced to consider these research aspects and allocate strategies and resources for them. There are various reasons for this neglect; still, factors such as lack of suitable instruments for detection and diagnosis, lower levels of awareness and practiced stigma affecting demand for ASD caregiving and the dominance of specialist models for diagnosis and treatment, and finally, the high cost of researching ASD contributed to this imbalance in LMICs [6]. There are understudied groups in the ASD population, such as groups with ASD and other developmental disabilities [7], and aspects of the life of individuals with ASD, such as their sexual development [8] in HICs.

It seems that healthcare priorities in LMICs force the service providers to focus on more fatal conditions such as infant mortality, AIDS, and, more recently, pandemic issues such as COVID-19 [9]. The reported orientation towards COVID-19 is not limited to LMICs, and HICs also reported a similar shift [10]. Hence, even before the COVID-19 pandemic, there was a shortage of awareness regarding ASD and its associated conditions in most LMICs. Some services started in some countries, but even when some sort of screening or diagnosis services is available, it is hampered by different factors such as the cost of the scale, extensive training to use some of the gold standard scales, and administrating issues [6] such as the copyright issues. The healthcare challenges in these societies might be reviewed from different aspects.

When the situation of individuals with ASD is reviewed in LMICs and among immigrants and minorities in HICs considering social aspects of healthcare is inevitable. Social issues in healthcare are social determinants of caregiving services in society [11] and are influenced by factors such as the dominant healthcare policies or different healthcare issue priorities. This situation is partly a result of a shortage of epidemiological and service research in mental health in general and ASD in particular in the LMICs. Nevertheless, the demographics of the worldwide human population are skewed toward HICs. It is estimated that the majority of world children and youth live in LMICs (up to 80%). It is also predicted that over 250 million children younger than five years old are at risk of not reaching their developmental potential and need healthcare services in LMICs [12,13]. On the other hand, findings reported barriers to equal access and support for immigrant families of individuals with ASD in HICs [14]. The contributing factors noted are cultural expectations about child development and ASD delayed diagnosis because of the long waiting lists, issues with services access, and negative perceptions of services [15].

There are different ASD prevalence reports among immigrants or refugee groups compared to the information from their original countries [16]. It might be the impact of discrepancies between the cultural expectations and social norms and the application of the available scales developed in HICs. Dominant ideas about development and behavior may impact understanding the signs or symptoms of a complex condition such as ASD. For example, there are societies where establishing eye contact between a child and an adult is considered inappropriate, or for a particular gender, it may be regarded as a positive sign. It is doubtful that having no eye contact draws the caregiver’s attention [4,17]. It means that diagnostic criteria and tools based on HICs’ norms of child behavior may be applied with higher degrees of caution for other cultures. Therefore, it is essential to consider the cultural context while providing diagnostic services for ASD in LMICs. Similar cultural issues have been reported regarding the diagnosis of ASD and its functional level among minorities in HICs [18]. It is also said that minority and immigrant children, most expatriates from LMICs, are less likely to receive on time a diagnosis of ASD [19]. They are also at risk of being overdiagnosed [20] because of the utilization of culturally biased scales and standards. Delayed diagnosis is a crucial issue regardless of the country’s development level. However, there are data regarding the diagnosis delay among immigrant children from LMICs to HICs. Still, a dearth of investigation on the ways that increase mutual understanding and collaboration between the healthcare providers in HICs and the immigrant population is considered to be the core of this delay [21]. Still, some reviews reported a considerable delay in diagnosis despite the early emergence of ASD symptoms without significant statistical differences between LMICs and HICs [22]. Individuals from high socioeconomic families are mainly considered in ASD research within HICs. For example, in North America, ASD research is often associated with stereotypes of Caucasians, well-to-do socioeconomic status, and masculinity. Expanding cross-cultural and autism-friendly scholarly attention to the topic is challenging without considering different cultures and predominantly on the broader world of ASD research. It means that culture is something others (Brazilians, Indians, Italians, etc.) “have” and not a solely “default” white Anglo-Saxon person [23,24,25,26].

There is a documented dearth of ASD studies among immigrants and minority groups in HICs [27]. Hence, the evidence supports ethnic and racial variances in ASD prevalence and access to services, even among the HICs and developed societies. There is also a focus on particular age groups of individuals with ASD. The skewness of the research on children caused a public perception of ASD as a childhood disability that fades through adulthood. The reason is that ASD tends to be identified and supported in the public educational system. Most available data to date have been obtained from and focused on children [28].

Another social factor that impacted ASD in most LMICs is the issue of stigma. The fear of being stigmatized may cause caregivers in some societies to deny the emergence of the symptoms. Especially at the first stages of symptom emergence, the signs seem more tolerable, particularly for children with high-functioning who are likely to be overlooked [29].

More attempt is needed from different shareholders in ASD to address the universal imbalance in knowledge on ASD in LMICs and among immigrants and minority groups in HICs. Social media such as Facebook helped international advocates coordinate efforts to tackle ASD worldwide [30]. Resolutions issued by the United Nations [31] and World Health Organization [32] and the “Autism Speaks” ‘Global Autism Public Health’ (GAPH) initiative [33] are some recent vital indicators of the importance of this global initiative. Sustainable progress in healthcare services development in ASD, similar to other mental healthcare services in LMICs, depends on considering politics, leadership, planning, advocacy, and participation [34].

This Special Issue might be considered another initiative to help understand ASD among different groups of people who might have been less represented. We aim to review the current knowledge about ASD differences, including the complex and multilayered impacts in LMICs and among immigrants and minority groups in high-income countries. We also aim to highlight areas in which further research is needed.
